# Erythromycin Estolate Is a Potent Inhibitor Against HCoV-OC43 by Directly Inactivating the Virus Particle

**DOI:** 10.3389/fcimb.2022.905248

**Published:** 2022-07-07

**Authors:** Xiaohuan Wang, Yongkang Chen, Huichun Shi, Peng Zou

**Affiliations:** ^1^ Shanghai Public Health Clinical Center, Fudan University, Shanghai, China; ^2^ Department of Laboratory Medicine, Zhongshan Hospital, Fudan University, Shanghai, China; ^3^ Shanghai Institute of Infectious Disease and Biosecurity, Fudan University, Shanghai, China

**Keywords:** erythromycin estolate, human coronavirus strain OC43 (HCoV-OC43), antivirals, inactivator, viral inhibitor

## Abstract

In addition to antibacterial effects, macrolide antibiotics exhibit other extensive pharmacological effects, such as anti-inflammatory and antiviral activities. Erythromycin estolate, one of the macrolide antibiotics, was previously investigated to effectively inhibit infections of various flaviviruses including Zika virus, dengue virus, and yellow fever virus, but its antiviral effect against human coronavirus remains unknown. Thus, the current study was designed to evaluate the antiviral efficacy of erythromycin estolate against human coronavirus strain OC43 (HCoV-OC43) and to illustrate the underlying mechanisms. Erythromycin estolate effectively inhibited HCoV-OC43 infection in different cell types and significantly reduced virus titers at safe concentration without cell cytotoxicity. Furthermore, erythromycin estolate was identified to inhibit HCoV-OC43 infection at the early stage and to irreversibly inactivate virus by disrupting the integrity of the viral membrane whose lipid component might be the target of action. Together, it was demonstrated that erythromycin estolate could be a potential therapeutic drug for HCoV-OC43 infection.

## Introduction

Coronaviruses (CoVs), which belong to the family of Coronaviridae, are enveloped, single-stranded, and positive-sense RNA viruses and characterized with a large genome of approximately 30 kb in length (Cui et al., 2019). According to their genome sequences and phylogenetic relationships, CoVs are subdivided into four genera: alpha (α)–, beta (β)–, gamma (γ)–, and delta (δ)–CoVs. Viruses in the genus of α- and β-CoVs cause respiratory or gastrointestinal infections in humans and animals, and human CoVs (HCoVs) are composed of two α-CoVs (HCoV-229E and HCoV-NL63) and five β-CoVs (HCoV-OC43, HCoV-HKU1, SARS-CoV, MERS-CoV, and recently emerging SARS-CoV-2) (Artese et al., 2020). These HCoVs pose a heavy disease burden and serious threat to public health.

Over the past 20 years, HCoVs have received global attention for their unprecedented outbreaks in community and hospitals. In 2002–2003, a severe respiratory disease caused by SARS-CoV outbroke and spread to 29 countries and regions within half a year, resulting in approximately 10% mortality (Su et al., 2016; Zumla et al., 2016). In 2012, cases with MERS-CoV infection were reported, which caused high mortality of approximately 30% (Zaki et al., 2012). Recently, a new strain of HCoV, named SARS-CoV-2, broke out in 2019 and rapidly spread worldwide, leading to the long-term global pandemic and severe burden on public health and economy (Chen et al., 2020; Wu and McGoogan, 2020). Meanwhile, some HCoVs strains, including 229E, NL63, and OC43, usually, not only caused mild upper respiratory tract infection, similar to a self-limiting common cold, but also contributed to some severe and even fatal complications in some groups, such as the children, elders, and immunocompromised patients (Simon et al., 2007; Walsh et al., 2013; Mayer et al., 2016; Morfopoulou et al., 2016).

Since the outbreak of coronavirus disease 2019 (COVID-19), a lot of candidate drugs against CoV have been developed, but only a few achieved the expected efficacy (Xu et al., 2021). Molnupiravir was urgently approved by the Food and drug administration (FDA) for the oral treatment of COVID-19, but the potential risk probably arising from its antiviral mechanism cannot be ignored (Dyer, 2021; Jayk Bernal et al., 2021). Thus, the *de novo* development of specific agents targeting HCoVs remains challenging. Moreover, drug repurposing, characterized with rapidness, safety, and efficiency, could be an alternative strategy to identify effective candidates for combating the challenging infection (Pushpakom et al., 2019). Some macrolide antibiotics have been investigated to effectively inhibit the infection of CoV as well. For example, azithromycin was tested to exhibit antiviral activity against SARS-CoV-2 in different points of viral cycle *in vitro* (Echeverría-Esnal et al., 2021). In addition, bafilomycin A1, clarithromycin, and lexithromycin also showed potential inhibitory activity against SARS-CoV-2 infection (Galvez et al., 2021; Shang et al., 2021).

Erythromycin estolate (Ery-Est), one of macrolide antibiotics, was previously demonstrated to effectively inhibit infections of various flaviviruses including Zika virus (ZIKV), dengue virus (DENV), and yellow fever virus (YFV) (Wang et al., 2019), but its antiviral effect against HCoVs remains unknown. Therefore, the antiviral activity of Ery-Est against HCoV-OC43 was studied, and the possible mechanism underlying its antiviral activity was investigated in this study.

## Materials and Methods

### Cells, Viruses, and Compounds

RD (rhabdomyosarcoma) cells, HCT-8 (colon carcinoma) cells, BHK-21 (Baby Hamster Kidney) cells, and Vero (African green monkey kidney) cells were obtained from American Type Culture Collection (ATCC; Manassas, VA, USA) and cultured in Dulbecco’s modified Eagle’s medium (DMEM; Biological Industries, Israel) containing 10% fetal bovine serum (FBS; Biological Industries, Israel) and supplemented with penicillin (100 U/ml) and streptomycin (100 μg/ml) at 37°C with 5% CO_2_.

HCoV-OC43 (VR-1558) obtained from ATCC and the enterovirus 71 (EV71), which are kindly provided by Dr. Shuye Zhang, were both propagated in RD cells. The viral supernatant was collected and centrifuged to remove cell debris and stored at −80°C.

Ery-Est, erythromycin (Ery), ebselen, and chloroquine phosphate powders were purchased from Santa Cruz Biotechnology, Inc. (Dallas, TX, USA), MedChemExpress (Monmouth Junction, NJ, USA) and Sigma-Aldrich (St. Louis, MO, USA), respectively. In addition, the lyophilized powder was dissolved in dimethyl sulfoxide (DMSO) and stored at −20°C, except that chloroquine phosphate was dissolved in sterilized water.

### Plaque Assay

Viral titer of HCoV-OC43 was determined on BHK-21 cells by plaque assay as previously described (Wang et al., 2019). Briefly, BHK-21 cells were cultured in cell culture plate at 37°C with 5% CO_2_ overnight to form confluent monolayer. Serially diluted viruses were added to cells and incubated for 2 h. Then, the inoculum was replaced with an overlay medium of DMEM that included 2% FBS and 0.6% LMP (low melting point) agarose (Promega Co., Madison, WI, USA). The infected cells were further incubated for approximately 4 days until the plaque was developed. Finally, the cells in wells of culture plate were fixed and stained with 4% formaldehyde and 1% crystal violet, respectively, for plaque visualization. Viral titer was expressed as plaque-forming units (PFU)/ml.

Ery-Est and Ery were serially diluted and then mixed with HCoV-OC43 or EV71. After incubation at 33°C or 37°C, respectively, for 1 h, the mixture was added to monolayer of BHK-21 cells (HCoV-OC43) or Vero cells (EV71) that seeded in cell plates and incubated for 2 h. Then, the supernatant was removed and replaced with DMEM containing 0.6% or 1% LMP agarose and 2% FBS. The plaque visualization was carried out with 4% formaldehyde and 1% crystal violet as described above when the viral plaques clearly developed.

### Drug Cytotoxicity Assay

RD cells and HCT-8 cells were seeded in 96-well plates with the density of 2 × 10^4^ cells per well and incubated at 37°C with 5% CO_2_ overnight. Following addition of serially diluted Ery-Est and Ery starting from 100 µM into cells and further incubation for 3 days, Cell Counting Kit-8 (CCK8; Dojindo, Japan) was then used to detect cell viability according to the reagent instruction manual. After the absorbance at 450-nm wavelength was measured by iMarkTM microplate reader (Bio-Rad, USA), the CC_50_ (50% cytotoxicity concentration) value was determined.

### Immunofluorescence Staining Assay

Cells were infected with HCoV-OC43 at multiplicity of infection (MOI) of 0.01, which was preincubated with serial diluted Ery-Est (10, 5, and 2.5 µM) or Ery (10 µM) for 1 h. The inoculum was removed and replaced by DMEM with 2% FBS at 2 h after infection. About 48 h later, cells were washed and then fixed and permeabilized with 4% paraformaldehyde and 0.2% Triton X-100, respectively. After blocking with 3% BSA (Amresco, Solon, OH, USA) in PBS, the mouse anti-HCoV-OC43 nucleoprotein mAb (Millipore, Temecula, CA, USA) and Fluorescein isothiocyanate (FITC)-labeled rabbit anti-mouse Immunoglobulin G (IgG) (1:500, Dako, Glostrup, Denmark) were applied to stain the HCoV-infected cells. After extensive washing, the cells were mounted in the Prolong Gold Antifade reagent with 4,6-diamidino-2-phenylindole (DAPI) (Thermo Fisher Scientific, Waltham, MA, USA) before observing under confocal microscope (Leica SP8).

### Assay for Antiviral Activity

RD cells or HCT-8 cells seeded in the cell plates were incubated overnight and then infected with HCoV-OC43 at an MOI of 0.1 or 1, respectively, which was pretreated with serial concentrations of Ery-Est. The supernatants were collected at 48 h after infection, and their viral titers were detected by plaque assay as described above. Ery was included as control.

### Time-of-Addition Assay

To investigate at which time Ery-Est exerted its inhibitory activity against HCoV-OC43, the time-of-addition assay was performed as previously described (Wang et al., 2019). BHK-21 cells were infected with 100 PFU of HCoV-OC43 and were treated with Ery-Est (5 µM) at different times (0, 1, 2, 4, 8, and 10 h) after infection. After 12-h incubation, BHK-21 cells were washed and then covered by DMEM with 2% FBS and 0.6% LMP agarose. The plaques of infected cells were visualized by 4% formaldehyde and 1% crystal violet as described above.

### Assays for Virus Entry, Post-Entry, Adsorption, and Internalization

To identify which step of viral life cycle the drug would disturb, assays for virus entry, post-entry, adsorption, and internalization were performed as previously described (Si et al., 2018; Wang et al., 2019). For the entry assay, BHK-21 cell monolayers were first infected by HCoV-OC43 at the presence of Ery-Est for 2 h and then were washed to remove unbound drugs and viruses. In post-entry assay, after the cells were infected with HCoV-OC43 for 2 h, Ery-Est was added to cells and incubated for 12 h. For the adsorption assay, cells were first infected with mixture of HCoV-OC43 and Ery-Est for 1 h at 4°C, the inoculums were then discarded, and cells were washed to remove unbound drugs and viruses. For internalization assay, after cells were infected with HCoV-OC43 for 1 h at 4°C and the unbound viruses were washed away, infected cells were incubated at 33°C with the treatment of Ery-Est for 1 h and then washed to remove drugs. Finally, the cells were covered with overlay medium (DMEM containing 2% FBS and 0.6% LMP agarose). The plaques were visualized by 4% formaldehyde and 1% crystal violet as described above.

### Pre-Treatment Assay

To test whether the target of antiviral effect of Ery-Est was cells or not, the pre-treatment experiment was performed as previously described (Lee et al., 2019). Ery-Est (5 µM) was added to BHK-21 cell monolayers and then incubated for 2 h, followed by extensive washing with PBS to remove drugs. BHK-21 cell monolayers treated with Ery-Est 2 h before infection and without removing the drug during infection were also included. Then, cells were infected with HCoV-OC43 at 33°C for 2 h and covered with overlay medium. Plaques of infected cells were visualized as described above.

### Assay to Detect Inactivated Virions

Whether Ery-Est could inactivate viral particles of HCoV-OC43 was detected as previously described (Wang et al., 2019). Simply, serial concentrations of Ery-Est were mixed with 3 × 10^3^ PFU of HCoV-OC43 and incubated for 2 h, followed by adding PEG-8000 (Amersco) and NaCl with the final concentrations of 10% and 0.67 M, respectively. Then, the mixture was incubated on ice for 2 h. After centrifugation at 20,200*g* for 1 h at 4°C and removing supernatant, 3% PEG-8000 in PBS with BSA (10 mg/mL) was added to wash the pellet. The viral particles in the pellet were resuspended after centrifugation again, and the infectious HCoV-OC43 particles were finally detected by plaque assay as described above.

### Infectivity Inhibition Reversibility Assay

The infectivity inhibition reversibility assay was performed as previously described (Lok et al., 2012). Simply, 100 PFU of viruses were incubated with 5 µM Ery-Est for 1 h in DMEM, followed by 100-fold dilution with DMEM. The diluted virus–drug mixtures containing 0.05 µM Ery-Est were immediately added to BHK-21 cell monolayers and incubated for 2 h at 33°C. Meanwhile, viruses with constant treatment of 5 µM and 0.05 µM Ery-Est were included as control. The infectivity of the treated HCoV-OC43 was determined by plaque assay as described above.

### RNase Digestion Assay and RT-qPCR

To test whether Ery-Est could induce genomic RNA release of HCoV-OC43, RNase digestion assay and RT-qPCR were performed as previously described (Wang et al., 2019). Serial concentrations of Ery-Est and Ery were incubated with HCoV-OC43 for 2 h, and micrococcal nuclease (New England BioLabs, Ipswich, MA, USA) was then added to the mixture and incubated for 2 h at 37°C to digest the released RNA from treated viruses. After the inactivation of RNase, the EasyPure Viral DNA/RNA Kit (Transgen Biotech, Beijing, China) was used to extract genomic RNA of integral viruses, which was then detected with TransScript II Green One-Step qRT-PCR SuperMix (Transgen Biotech, Beijing, China) by a Master Cycler Ep Realplex PCR system (Eppendorf, Hamburg, Germany) according to the manufacturer’s instructions. Primers used in RT-qPCR to detect HCoV-OC43 RNA sequence coding viral nucleoprotein were as follows: F1 (5′-AGCAACCAGGCTGATGTCAATACC-3′)/R1 (5′-AGCAGACCTTCCTGAGCCTTCAAT-3′).

### Assays of Liposome Leakage

The liposomes containing fluorescent dye sulforhodamine B (Lip-SRB) were synthesized by Shanghai DDSome Lab Ltd. as previously described (Chen et al., 2013). Lip-SRB and drugs were diluted in isotonic physiological buffer (IPB) constituted with 140 mM NaCl and 17 mM HEPES (pH 7.4). Then, diluted Lip-SRB (5 μg/ml) were incubated with Ery-Est (80 μΜ and 40 μΜ) or Ery (80 μΜ), respectively, for 2 h at room temperature. Lip-SRB treated by Triton X-100 and DMSO were included as positive and negative control. The leakage of SRB was detected by Fluorescence Microplate Reader (BioTek, Vermont, USA) at the excitation and emission wavelength of 490 nm and 570–610 nm, respectively.

### Statistical Analysis

All data were analyzed by using the software of GraphPad Prism 6.0 (GraphPad Software, Inc.). Data were represented as means ± SD. In addition, * *p* < 0.05, ** *p* < 0.01, *** *p* < 0.001, and **** *p* < 0.0001 were considered as significant difference.

## Results

### Erythromycin Estolate Effectively Inhibited the Infection of HCoV-OC43 in Different Host Cells

**Figure 1:**
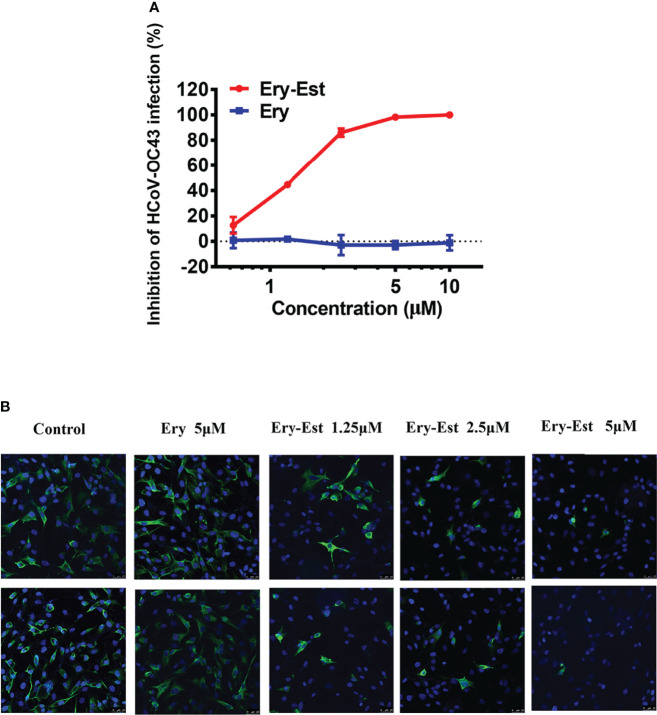
The anti-HCoV-OC43 activity of Ery-Est. **(A)** Ery-Est suppressed HCoV-OC43 infection at a dose-dependent manner by plaque reduction assay using BHK-21 cells. The experiment was performed in triplicate, and the data were presented as means ± SD. **(B)** The anti-HCoV-OC43 effect of Ery-Est detected by immunofluorescence assay. HCoV-OC43 nucleoprotein was stained by anti-nucleoprotein mouse mAb and FITC-conjugated rabbit anti-mouse IgG (green); nuclei (blue) were stained by 4,6-diamidino-2-phenylindole (DAPI).

Both Ery and Ery-Est are macrolide antibiotics used for the treatment of a variety of bacterial infections, and the latter is the lauryl sulfate ester of propionyl Ery. It was reported previously that Ery-Est has the ability to block infection of flaviviruses, such as ZIKV and DENV. Plaque reduction assay, a method by which the inhibitory effect of drugs could be determined visually, was performed to detect the antiviral activity of Ery-Est and Ery against HCoV-OC43 infection. It was showed that Ery-Est could effectively inhibit infection of HCoV-OC43 with the 50% inhibitory concentration (IC_50_) value of 1.40 ± 0.08 µM (**Figure 1A**). In addition, immunofluorescence staining assay was also employed to test the anti-HCoV-OC43 activity of Ery-Est at three different concentrations. As shown in **Figure 1B**, the number of HCoV-OC43-infected cells decreased as the concentration of Ery-Est increased, and the nucleoprotein of HCoV-OC43 was barely detected with the treatment of 5 µM Ery-Est. The treatment of 5 µM Ery did not show antiviral effect against HCoV-OC43 infection in plaque reduction assay or in immunofluorescence staining assay.

**Figure 2:**
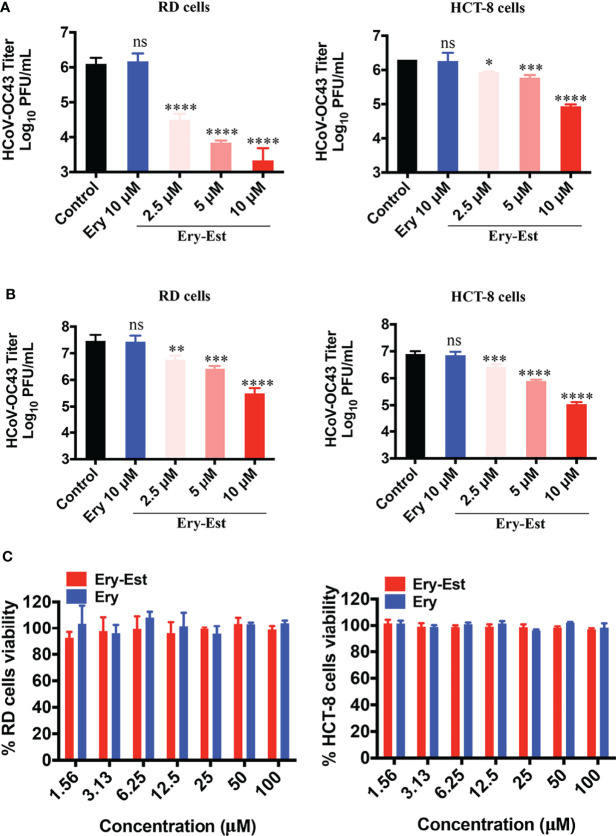
Ery-Est reduced HCoV-OC43 titers in supernatants of infected RD and HCT-8 cells. **(A)** The inhibitory effect of Ery-Est on supernatant titers at an MOI of 0.1. **(B)** The inhibitory effect of Ery-Est on supernatant titers at an MOI of 1. **(C)** Ery-Est showed no cytotoxic to RD cells and HCT-8 cells. Each experiment was performed in triplicate, and the data were presented as means ± SD. ns, not significant; *p < 0.05; **p < 0.01; ***p < 0.001; ****p < 0.0001.

Moreover, to further determine whether Ery-Est could inhibit HCoV-OC43 infection in human cell lines, RD cells and HCT-8 cells were infected with HCoV-OC43 at an MOI of 0.1 or 1, respectively, with the treatments of serial concentrations of Ery-Est (2.5, 5, and 10 µM) and Ery (10 µM). Because RD cells and HCT-8 cells are not suitable for performing plaque reduction assay, the viral titer in the supernatant was determined by plaque assay using BHK-21 cells. Ery-Est significantly reduced HCoV-OC43 titers at the concentration of 2.5 µM, whereas Ery treatment almost exhibited no inhibition even at 10 µM (**Figures 2A, B**). In addition, Ery-Est and Ery exhibited no obvious toxicity to RD and HCT-8 cells at 100 µM (**Figure 2C**). These results indicated that Ery-Est robustly suppressed HCoV-OC43 infection in different host cells.

### Erythromycin Estolate Inhibited HCoV-OC43 Infection at the Early Stage

**Figure 3:**
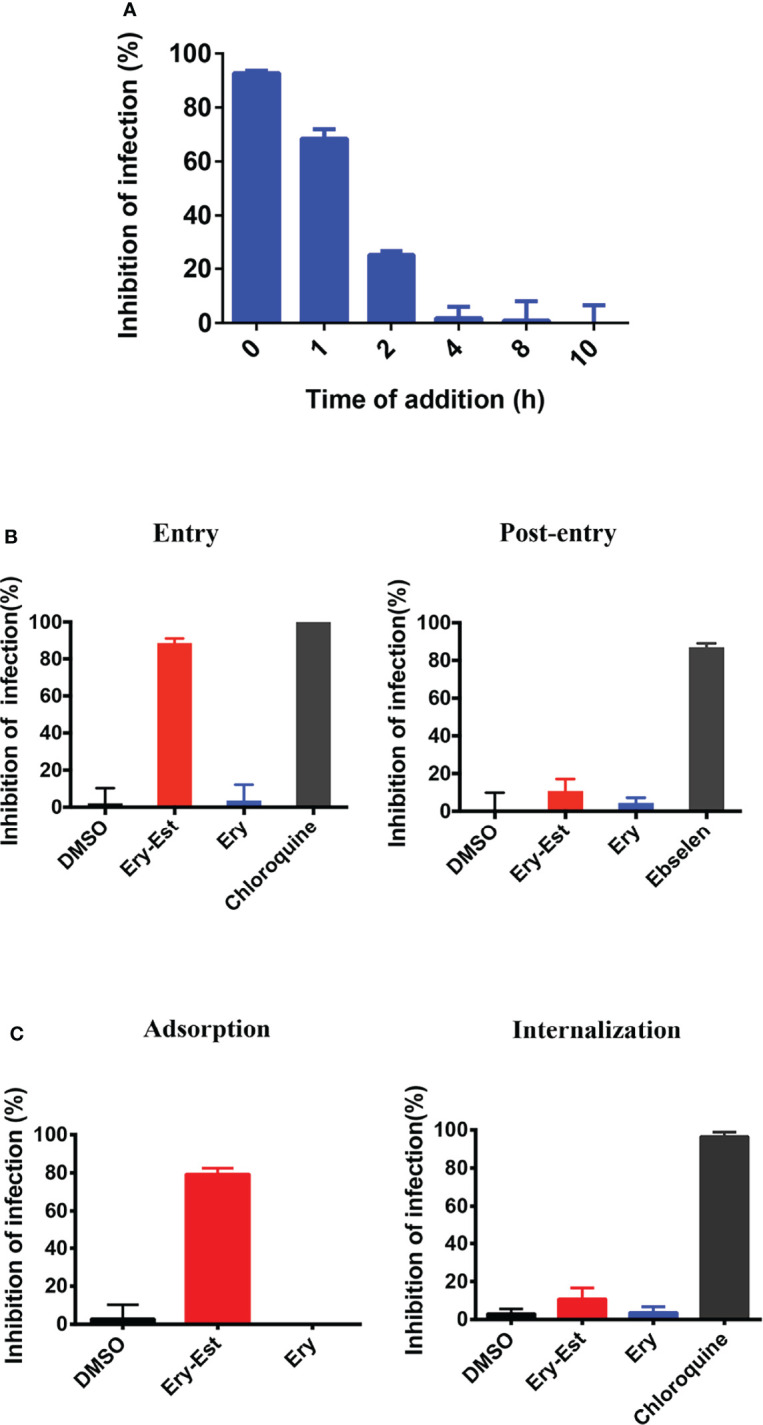
Ery-Est inhibited HCoV-OC43 infection at the early stage. **(A)** Time-of-addition assay showed inhibitory activity of Ery-Est within 2 h after HCoV-OC43 infection. **(B)** Ery-Est suppressed virus entry but barely affected the post-entry stage. **(C)** Ery-Est disturbed the step of attachment but had not impact on viral internalization. Each experiment was performed in triplicate, and the data were presented as means ± SD.

Ery-Est was previously verified to block ZIKV infection at the early stage of virus life cycle (Wang et al., 2019). Thus, the time-of-addition assay was first performed to determine at which stage HCoV-OC43 infection was susceptible to Ery-Est inhibition. Ery-Est (5 µM) was added to inoculum at different time points after virus infection; plaque assay was then used to assess the inhibitory effects. As shown in **Figure 3A**, Ery-Est exerted significant antiviral activity against infection of HCoV-OC43 at the beginning of infection. The inhibitory activity gradually decreased when Ery-Est was added later, and Ery-Est barely inhibited HCoV-OC43 infection since 4 h after infection. This suggested that Ery-Est inhibited HCoV-OC43 infection at the early stage of virus life cycle, mainly within the first 2 h of infection.

To further confirm the inhibitory behavior of Ery-Est on HCoV-OC43, experiments were employed as previously described, which divided the infection into two stages: entry and post-entry (Si et al., 2018; Wang et al., 2019). It was demonstrated that Ery-Est significantly suppressed viral entry but had no obvious effect at the post-entry stage (**Figure 3B**). Next, experiments that subdivided the process of viral entry into adsorption and internalization were individually performed to investigate the precise stage at which Ery-Est blocked infection of HCoV-OC43. **Figure 3C** showed that Ery-Est significantly disturbed the step of adsorption but had no impact on viral internalization. Overall, these results suggested that Ery-Est exerted antiviral effect as an entry inhibitor, particularly obstructing the adsorption stage.

### Erythromycin Estolate Irreversibly Inactivated Viral Particles and Induced Genomic RNA Release

**Figure 4:**
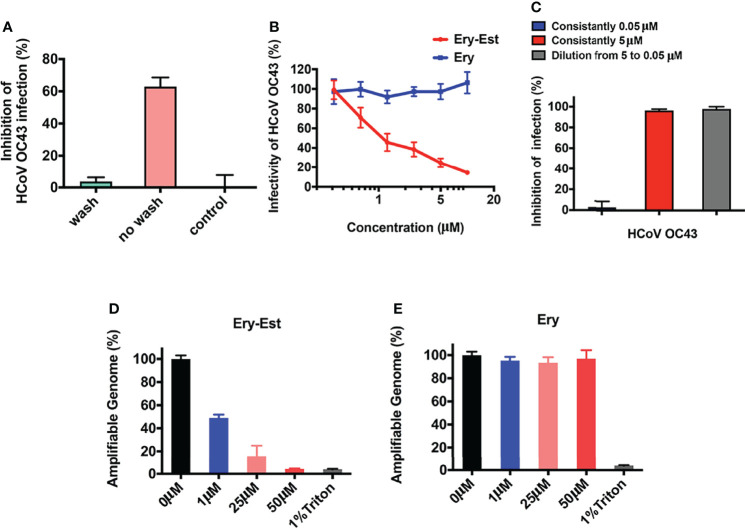
Ery-Est irreversibly reduced the infectivity of HCoV-OC43. **(A)** Ery-Est inhibited HCoV-OC43 infection only by keeping Ery-Est present during the virus infection. **(B)** Ery-Est reduced the infectivity of HCoV-OC43 at a dose-dependent manner after Ery-Est was separated from viruses by PEG-8000 precipitation. **(C)** Ery-Est irreversibly inactivated the infectivity of HCoV-OC43. **(D, E)** Ery-Est treatment but not Ery treatment caused the release of HCoV-OC43 genomic RNA. Each experiment was performed in triplicate, and the data were presented as means ± SD.

The results above demonstrated that Ery-Est blocked the attachment step of viral life cycle, in which both viruses and cells might be the inhibitory targets for drug treatment. Therefore, assays were conducted to make it clear. After incubation with Ery-Est for 2 h, cells were treated with washes to remove Ery-Est or without removing Ery-Est treatment, respectively, followed by infection of HCoV-OC43. It was demonstrated that the inhibition was achieved only by keeping Ery-Est present during the course of virus infection (**Figure 4A**), whereas removing Ery-Est before infection resulted in the loss of antiviral effect, implying that the inhibitory targets of Ery-Est were focused on viral particles.

Next, to confirm whether Ery-Est could reduce the viral infectivity of HCoV-OC43 directly, the assay was carried out to detect inactivation effect of drug as previously described (Wang et al., 2019), in which PEG-8000 was used to separate viral particles from Ery-Est before HCoV-OC43 infected cells. As shown in **Figure 4B**, the infectivity of HCoV-OC43 diminished even after it was separated from Ery-Est, indicating that Ery-Est has already inactivated viruses during incubation with the viruses. In addition, the infectivity gradually reduced as the concentration of Ery-Est increased. Then, whether the inactivation activity of Ery-Est on HCoV-OC43 was irreversible aroused our interest. According to the result that Ery-Est was sufficient to block viral infectivity at 5 µM and barely block infection of HCoV-OC43 below 0.3 µM, Ery-Est (5 µM) was incubated with HCoV-OC43 for 1 h and then diluted 100-fold, which resulted in the decline of Ery-Est concentration from 5 to 0.05 µM. The plaque assay was then performed immediately to test HCoV-OC43 infectivity. Results demonstrated that the infectivity of HCoV-43 did not recover after dilution of Ery-Est (**Figure 4C**), implying the irreversibility of Ery-Est inactivation on HCoV-OC43.

As Ery-Est could irreversibly inhibit infectivity of viral particles, potential mechanism of inactivation became an interesting question worthy of further research. Some viral inactivators, such as peptide Z2 and montelukast, were reported to break the integrity of viral particles, and Ery-Est was previously reported to induce the genomic RNA release of ZIKV (Yu et al., 2017; Chen et al., 2019; Si et al., 2019). An RNase digestion assay was thus used to detect genomic RNA release of HCoV-OC43, in which the exposure of viral genomic RNA due to damage of virion’s integrity caused by drug treatment would be sensitive to RNase digestion, but viral RNA in intact viral particles would be shielded from RNA degradation. **Figures 4D, E** showed that genomic RNA of HCoV-OC43 was gradually degraded and undetectable by Ery-Est treatment in a dose-dependent manner, and about half of viral RNA was exposed and digested at the concentrations of 1 µM, whereas viral RNA inside Ery-treated viruses was well protected from RNase digestion. It illustrated that Ery-Est may inactivate HCoV-OC43 by inducing the release of genomic RNA.

### Erythromycin Estolate Disrupted the Integrity of Liposome

**Figure 5:**
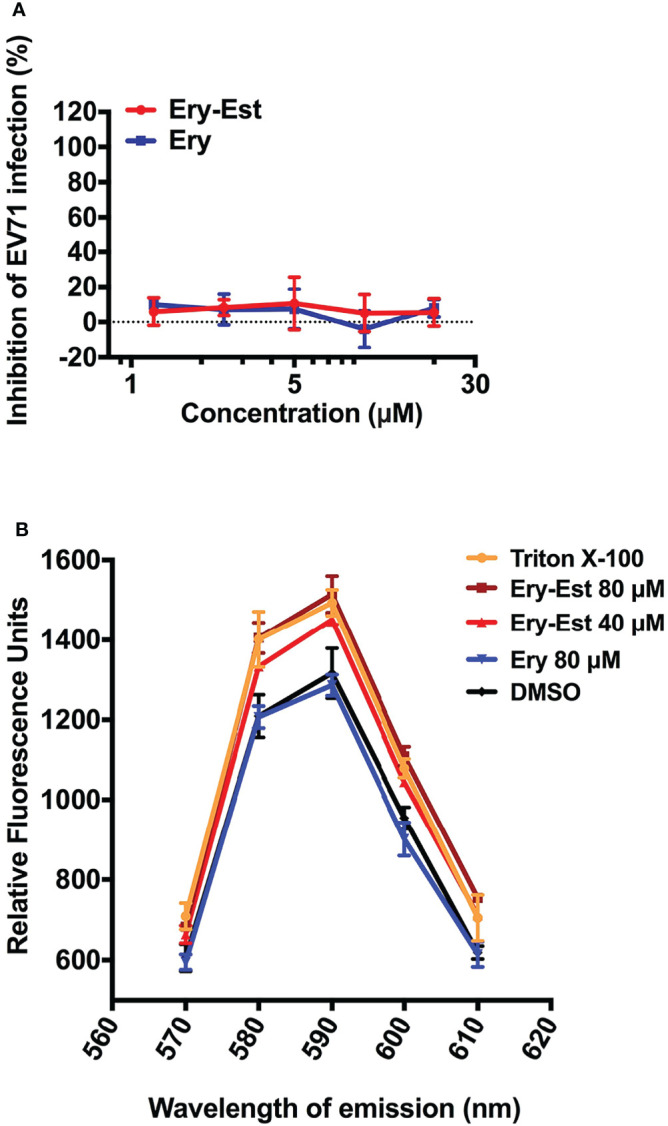
Ery-Est showed no inhibitory activity on non-enveloped virus EV-71 **(A)** and could disrupted the integrity of liposome encapsulating fluorescent dye SRB, leading to the increase of the fluorescence intensity **(B)**. Each experiment was performed in triplicate, and the data were presented as means ± SD.

Because Ery-Est was capable to inhibit infections of various enveloped viruses including HCoV-OC43 in this study and several kinds of flaviviruses reported previously, we examined whether Ery-Est could inhibit infection of non-enveloped virus or not. The plaque reduction assay was performed to detect the antiviral activity of Ery-Est and Ery against infection of EV71, a typical non-enveloped virus. As shown in **Figure 5A**, Ery-Est exhibited no inhibitory effect on EV71 infection, indicating that Ery-Est was not able to inhibit the infection of non-enveloped virus. On the basis of these results, we speculated that Ery-Est could probably act on the lipid, which is the main component of viral envelope. To simulate the lipid envelope of virus particles, liposome encapsulating fluorescent dye SRB was synthesized as previously described (Chen et al., 2013). The increase of fluorescence intensity of SRB could reflect the leakage of SRB if the liposome structure is damaged. Various concentrations of Ery-Est and Ery were incubated with liposomes (5 μg/ml), and the fluorescence intensity of SRB in each group was shown in **Figure 5B**. Compared with the DMSO-treated control, Ery treatment barely changed the fluorescence intensity, whereas the treatments of Ery-Est or Triton X-100 significantly increased fluorescence intensity. It suggested that Ery-Est destroyed the structural integrity of liposome to release its internal SRB, indicating that Ery-Est could break viral particle to release genomic RNA by acting on the lipid of the viral envelope, finally disabling the infectivity of the virus.

## Discussion

HCoVs are important pathogenic viruses that seriously threaten human public health. In recent years, the unexpected pandemic of COVID-19 caused hundreds of millions of people to tragically suffer from the disease. Therefore, it is of great significance to develop anti-HCoV drug candidates, and drug repurposing was widely applied in the development of antiviral agents. However, experiments involving highly pathogenic HCoVs, such as SARS-CoV, MERS-CoV, and SARS-CoV-2, need to be performed in biosafety level 3 (BSL-3) facilities, whereas the research institutions with BSL-3 facilities are relatively inadequate (Shahkarami et al., 2015). Owing to the similar biological characteristics among HCoV-OC43 and those highly pathogenic HCoVs and its weak pathogenicity, HCoV-OC43 has been used as an alternative strain for scientific research by some researchers (Kim et al., 2019; Min et al., 2020; Yang et al., 2020).

Ery-Est, one of the macrolide antibiotics, was previously found to significantly inhibit infections of ZIKV and other flaviviruses by the strategy of drug repurposing (Wang et al., 2019). In this study, the inhibitory activity of Ery-Est against HCoV-OC43 was investigated, and it was found that Ery-Est could effectively inhibit HCoV-OC43 infection in BHK-21 by plaque reduction assay, and viral titers in supernatant of HCoV-OC43–infected RD and HCT-8 cells were significantly reduced with 2.5 µM Ery-Est treatment. It was reported that the peak concentration of Ery-Est in plasma with multiple doses was about 5.93 ± 2.34 μg/ml (5.61 ± 2.22 μM) (Croteau et al., 1988), indicating that Ery-Est could achieve effective antiviral concentration in infected patients. It is very promising that Ery-Est could exert inhibitory activity against highly pathogenic HCoVs, such as SARS-CoV, MERS-CoV, and the newly emerging SARS-CoV-2, which need further investigation.

Macrolide antibiotics, represented by Ery, are orally active antibiotics that bind to bacterial ribosome and inhibit its function of protein synthesis. Moreover, some macrolide antibiotics have been reported to possess inhibitory activity against infection of a variety of viruses. Azithromycin, a macrolide antibiotic used for treating bacterial infection in lower respiratory tract, was found to block infection of rhinovirus, ZIKV, and influenza A virus by different antiviral mechanisms (Oliver and Hinks, 2021), such as by increasing rhinovirus-induced interferons and interferon-stimulated gene expression, hence reducing rhinovirus replication (Gielen et al., 2010; Porter et al., 2016), by interfering with influenza A virus internalization process (Tran et al., 2019) and significantly ameliorating inflammation (Lee et al., 2017), by upregulating ZIKV-induced type I and III interferon (IFN) responses, and by enhancing expression of pathogen recognition receptors MDA5 and RIG-I (Li et al., 2019). Other macrolide antibiotics, such as clarithromycin and bafilomycin A1, could also inhibit infection of influenza virus and respiratory syncytial virus (Asada et al., 2009; Yeganeh et al., 2015; Yamamoto et al., 2016; Arikata et al., 2019). Infections of CoVs, including SARS-CoV-2, were also suppressed by azithromycin, bafilomycin A1, clarithromycin, and lexithromycin (Galvez et al., 2021; Shang et al., 2021). The inhibitory effect of azithromycin against SARS-CoV-2 might involve multiple mechanisms. Through molecular dynamics simulations, similarity between azithromycin and the sugar moiety of ganglioside GM1 was found, indicating that azithromycin could probably interact with SARS-CoV-2 spike protein and interfere with binding of spike protein and ACE2 hence block virus entry into cells (Fantini et al., 2020). Moreover, azithromycin was reported to impair the acidification of lysosome/late endosome and reduce the activity of protease required for pH-dependent processing of spike protein, which ultimately hindered the uncoating of SARS-CoV-2 and releasing viral RNA into cells (Nujić et al., 2012; Hoffmann et al., 2020). In addition to the direct inhibition mentioned above, indirect antiviral activity of azithromycin to amplify the cellular antiviral response mediated by the IFN pathway may also play a role.

Therefore, the inhibitory mechanism of Ery-Est on HCoV-OC43 infection was also preliminarily explored. Ery-Est was demonstrated to directly disrupt the viral integrity of HCoV-OC43 probably by causing damage of viral lipid envelope, leading to the release of viral genomic RNA and the irreversible loss of viral infectivity in the early stage of HCoV-OC43 infection. The direct inactivation activity of Ery-Est enriched the types of antiviral mechanism of macrolide antibiotics. It also explains the relatively broad antiviral effect of Ery-Est on different enveloped viruses, including HCoV-OC43, ZIKV, DENV, and YFV. The inhibitory mechanism of Ery-Est is similar to that of a micromolecular agent CLR01, which was reported to inhibit a lot of enveloped viruses (Röcker et al., 2018; Brenner et al., 2021) and to selectively act on the lipid-rich raft region of the viral envelope, causing the virus to release genomic RNA and lose its infectivity (Lopes et al., 2015; Röcker et al., 2018).

It is interesting that Ery-Est was found to inactivate HCoV-OC43 by damaging the viral lipid envelope at the concentration without cytotoxicity. Coincidentally, peptide DN59, small molecular compound curcumin, and molecular tweezer CLR01 all inhibited infection of many enveloped viruses such as flaviviruses and Ebola virus by causing disruption of viral membrane while having no obvious effect on cell viability (Hrobowski et al., 2005; Lok et al., 2012; Chen et al., 2013; Röcker et al., 2018). The different susceptibility of viral membrane and cellular membrane to these antivirals may reflect the differences in the composition of lipid components between these two kinds of membrane (Röcker et al., 2018). The extracellular matrix, an organized shell of secreted macromolecules covering the cells, may also help to improve the resistance of cell membrane to these agents. In conclusion, considering that the C_max_ of Ery-Est ranged from 3.08 µg/ml (2.92 µM) to 5.93 µg/ml (5.61 µM) and it could last for 5 to 6 hours that the plasma concentration was greater than 2 µg/ml (1.89 µM) (Croteau et al., 1988), Ery-Est is thus a promising candidate for treatment of HCoV-OC43 infection.

## Data Availability Statement

The original contributions presented in the study are included in the article/supplementary material. Further inquiries can be directed to the corresponding author.

## Author Contributions

PZ conceived the idea and designed the experiments; XW performed the experiments and analyzed the data; YC assisted XW in performing the experiments; HS assisted XW in analyzing the data; XW wrote the draft of the manuscript; XW, YC, and PZ revised the manuscript. All authors contributed to the article and approved the submitted version.

## Funding

This work was supported by grant from the Shanghai Public Health Clinical Center (KY-GW-2017-17).

## Conflict of Interest

The authors declare that the research was conducted in the absence of any commercial or financial relationships that could be construed as a potential conflict of interest.

## Publisher’s Note

All claims expressed in this article are solely those of the authors and do not necessarily represent those of their affiliated organizations, or those of the publisher, the editors and the reviewers. Any product that may be evaluated in this article, or claim that may be made by its manufacturer, is not guaranteed or endorsed by the publisher.
